# Gene Expression Profile Provides Novel Insights of Fasting-Refeeding Response in Zebrafish Skeletal Muscle

**DOI:** 10.3390/nu14112239

**Published:** 2022-05-27

**Authors:** Takehito Sugasawa, Ritsuko Komine, Lev Manevich, Shinsuke Tamai, Kazuhiro Takekoshi, Yasuharu Kanki

**Affiliations:** 1 Laboratory of Clinical Examination and Sports Medicine, Department of Clinical Medicine, Faculty of Medicine, University of Tsukuba, 1-1-1 Tennodai, Tsukuba 305-8577, Ibaraki, Japan; take0716@krf.biglobe.ne.jp (T.S.); shinsuke.tamai@jpnsport.go.jp (S.T.); 2 Department of Sports Medicine Analysis, Open Facility Network Office, Organization for Open Facility Initiatives, University of Tsukuba, 1-1-1 Tennodai, Tsukuba 305-8577, Ibaraki, Japan; srs8414@u.tsukuba.ac.jp; 3 Doctoral Program in Sports Medicine, Graduate School of Comprehensive Human Sciences, University of Tsukuba, 1-1-1 Tennodai, Tsukuba 305-8577, Ibaraki, Japan; 4 Experimental Pathology, Faculty of Medicine, University of Tsukuba, 1-1-1 Tennodai, Tsukuba 305-8577, Ibaraki, Japan; lew.manewitsch@gmail.com; 5 Doctoral Program in Biomedical Sciences, Graduate School of Comprehensive Human Sciences, University of Tsukuba, 1-1-1 Tennodai, Tsukuba 305-8577, Ibaraki, Japan; 6 Department of Sport Science and Research, Japan Institute of Sports Sciences, 3-15-1 Nishigaoka, Kita-ku, Tokyo 115-0056, Japan

**Keywords:** fasting, refeeding, skeletal muscle, zebrafish, mRNA-sequencing, gene length

## Abstract

Recently, fasting has been spotlighted from a healthcare perspective. However, the de-tailed biological mechanisms and significance by which the effects of fasting confer health benefits are not yet clear. Due to certain advantages of the zebrafish as a vertebrate model, it is widely utilized in biological studies. However, the biological responses to nutrient metabolism within zebrafish skeletal muscles have not yet been amply reported. Therefore, we aimed to reveal a gene expression profile in zebrafish skeletal muscles in response to fasting-refeeding. Accordingly, mRNA-sequencing and bioinformatics analysis were performed to examine comprehensive gene expression changes in skeletal muscle tissues during fasting-refeeding. Our results produced a novel set of nutrition-related genes under a fasting-refeeding protocol. Moreover, we found that five genes were dramatically upregulated in each fasting (for 24 h) and refeeding (after 3 h), exhibiting a rapid response to the provided conditional changes. The assessment of the gene length revealed that the gene set whose expression was elevated only after 3 h of refeeding had a shorter length, suggesting that nutrition-related gene function is associated with gene length. Taken together, our results from the bioinformatics analyses provide new insights into biological mechanisms induced by fasting-refeeding conditions within zebrafish skeletal muscle.

## 1. Introduction

Inappropriate calorie intake is an important risk factor for cardiovascular disease, and metabolic syndrome [1]. In recent years, fasting and caloric restriction have been attracting a certain attention as a part of health maintenance [1,2,3,4,5,6]. For example, intermittent fasting, which is a regular energy restriction, can provide health benefits for the prevention or treatment of many chronic diseases, including diabetes, cardiovascular disease, cancer, and neurodegenerative diseases [3]. Previous studies reported the effects of fasting on rodent and human health and lifespan [1,2]. In one study, Goodrick et al. reported that the average lifespan of rats with intermittent feeding from 5 weeks of age increased by up to 80% [7]. However, the biological mechanisms by which fasting provides health benefits still leave many questions unanswered. In particular, even though skeletal muscle consumes significant energy and uses ketones during fasting conditions, the effects of fasting on skeletal muscle are not well understood at the molecular level.

The zebrafish offers several advantages as a model organism that mimics mammalian genetic and physiological responses [8,9,10,11]. The zebrafish is a popular animal model because of its simplified maintenance, rapid growth rate, ease of genetic manipulation, and fewer ethical restrictions [12]. Fasting and refeeding protocols are commonly used to investigate the biological response of teleosts such as the transition from catabolic to anabolic states [13,14,15,16,17,18,19,20]. Several studies in zebrafish have investigated the comprehensive gene expression response to starvation diets in several tissues [13,21,22,23]. However, as the molecular mechanisms of a response to a fasting-refeeding protocol remain complex, further investigation at the molecular level using zebrafish is needed. To date, there are no detailed reports on the metabolic changes within skeletal muscles in response to variable nutrient intake. Hence, in this study we aimed to reveal the gene expression profile during responses to fasting-refeeding in zebrafish skeletal muscle. Subsequently, we performed mRNA-sequencing and bioinformatics analyses to assess the gene expression profile under fasting-refeeding conditions. According to the results, our study provides new insights into biological mechanisms, representing immediate transcriptional responses to the fasting-refeeding approach in zebrafish; as well as proposing efficient methods of bioinformatics analysis accompanied by mRNA-sequencing analysis of zebrafish skeletal muscle.

## 2. Materials and Methods

### 2.1. Zebrafish Experiments

**Figure 1:**
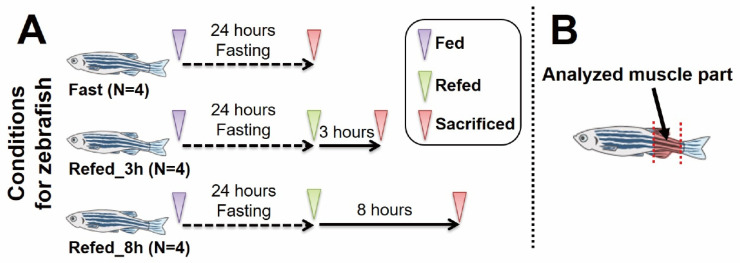
The overview of the experimental protocol. (**A**) The timing of experimental interventions in each group. (**B**) The location of the collected skeletal muscle tissue.

Zebrafish experiments in this study were approved by the Animal Care Committee, University of Tsukuba (approval number: 21-406). The overview of this experimental protocol is shown in Figure 1. Three-month-old zebrafish, of male/female ratio of approximately 1:1, were purchased from a local vendor (MASUKO; Kuki, Saitama, Japan), and then subjected to an acclimation period of over a month. During the acclimation period, the fish were maintained in a 15 L water tank with water temperature of 28.5 ± 1.0 °C and were given standard food for tropical fish (Tetra plankton; Spectrum Brands Japan, Yokohama, Kanagawa, Japan) twice a day every 12 h. The composition of this standard food is described by the manufacturer as follows: crude protein: 44.0%; crude fat: 11.0%; crude fiber: 2.0%; crude ash: 9.0%; moisture: 8.0%. Each feeding was an amount that the fish could eat within 2 min. After the acclimation period, 18–20 fish (gender ratio 1:1 approximately) were placed in a small 3.5 L water tank with water temperature of 28.5 ± 1.0 °C and subjected to a 24 h acclimation period being supplied with standard food. Next, fish were transferred to a small water tank including 380 mg of the same food. Then, four randomly selected fish (two female and two male) were sacrificed at the following three time points to make each condition. After the feeding, the fish were deprived of food for 24 h to impose a fasting condition (Fast). Subsequently, the other fish were refed using the same amount of food as previously. At 3 and 8 h following the refeeding, fish were sacrificed to produce conditions of refeeding after 3 and 8 h (Refed_3h and Refed_8h, respectively; Figure 1A). In each condition, a muscle tissue was harvested between the anus and caudal tail fin (Figure 1B), and was placed on ice for further RNA extraction. Average weight (mg) and standard error (SE) for all zebrafish (*N* = 12, in total) used in this study was 588.7 ± 27.2.

### 2.2. RNA Extraction

Total RNA was extracted from the tissues, using RNAiso plus (Cat#9108; Takara Bio, Kusatsu, Shiga, Japan), according to the manufacturer’s instructions. The extracted total RNA solution in Milli-Q water was diluted and adjusted to a concentration of 100 ng/μL. Then, 500 ng of RNA was used to make cDNA with the PrimeScript RT Master Mix (Cat#RR036A, Takara Bio), according to the manufacturer’s instructions. The cDNA was diluted 10 times by Milli-Q water for further analysis.

### 2.3. mRNA-Sequencing

The mRNA-sequencing (mRNA-seq) was performed to identify the whole gene expression profile in the tissues upon the fasting and refeeding conditions. The mRNA-seq analysis was conducted at the Department of Sports Medicine under the Open Facility Network Office, University of Tsukuba (Tsukuba, Ibaraki, Japan). A total of 12 RNA samples including *N* = 4 for each condition (i.e., Fast, Refed_3h, and Refed_8h) were examined for integrity using an Agilent RNA 6000 Nano Kit (Cat# 5067-1511; Agilent Technologies, Santa Clara, CA, USA) on a Bioanalyzer (Agilent Technologies). After the examination of the RNA Integrity Number, the RNA of all 12 samples was subjected to library preparations for mRNA-seq. Using 1.25 µg of the total RNA from each sample, libraries were created using the NEBNext Ultra II RNA Library Prep Kit for Illumina and the NEBNext Poly(A) mRNA Magnetic Isolation Module (Cat# E7770 and E7490; New England Biolabs, Ipswich, MA, USA), according to the manufacturer’s instructions; the final PCR cycle was 12. Concentration and size distributions of the libraries were measured using an Agilent DNA 7500 kit (Cat# 5067-1506; Agilent Technologies) with a Bioanalyzer. All samples were passed for analyses on next-generation sequencing (NGS) equipment. The libraries were pooled, and the concentrations adjusted to 1 nM. The pooled libraries were subjected to denaturation and neutralization. Subsequently, the libraries were diluted to 1.8 pM and then applied for an NGS run using NextSeq 500/550 v2.5 (75 cycles) kits (Cat#20024906; Illumina, San Diego, CA, USA) in a NextSeq 500 System (Illumina). The sequencing was performed with paired-end reads of 36 bases. After the sequencing run, FASTQ files were exported, and the basic information of the NGS run data was checked by CLC Genomics Workbench 20.0.4 software (QIAGEN, Venlo, Limburg, The Netherlands). In the quality assessment of the reads, a PHRED score over 20 was confirmed for 99.73% of all reads, indicating the success of the run. The read number was approximately 32.1 million to 49.1 million per sample as paired-end reads.

### 2.4. Bioinformatics Analysis

The FASTQ files were mapped to the zebrafish genome (*Danio rerio*; GRCz11) and expression values of all genes were obtained as TPM (Transcripts Per Kilobase Million) using the CLC Genomics Workbench software (Table S1). Statistical differential expression analysis was conducted using the “Differential Expression for mRNA-Seq” tool in the CLC. Differentially expressed genes (DEGs) were considered with a false discovery rate (FDR) < 0.05 and a two-fold change cutoff. For further analyses on the DEGs, to avoid the possibility of a sample’s contamination during the skeletal muscle extraction, genes with a standard deviation greater than 1 (TPM values of each gene were normalized to 1) were excluded in each group. Additionally, genes with an absolute average TPM value of less than 10 were considered as genes with a low expression and excluded from the analysis. A principal component analysis (PCA) plot and Venn diagram were created using the CLC software. Transcripts per kilobase million (TPM) were used as an expression value for figure visualizations. The Database for Annotation, Visualization, and Integrated Discovery web tool (DAVID version 6.8; Laboratory of Human Retrovirology and Immunoinformatics (LHRI), Frederick, MD, USA, https://david.ncifcrf.gov/; accessed on 28 June 2021) was used for enrichment analysis with default settings. Public databases of UniProtKB (https://www.uniprot.org/; accessed on 21 April 2022) and ZFIN (https://zfin.org/; accessed on 21 April 2022) were used to investigate function profiles of selected DEGs. The gene length and number of genes used the value annotated in the expression tracks of the CLC software. A heat map with clustering analysis, and box plots were generated using the CLC software, Jupyter Notebook (version 6.1.4), and Python (version 3.8.5). A cluster dendrogram with Ward’s minimum variance method was generated using R software (version 4.1.1). Plot graphs were created using GraphPad Prism software (version 9.3.1; GraphPad, San Diego, CA, USA). The scatter plot was created using Excel (Office 2019; Microsoft, Redmond, WA, USA).

### 2.5. Statistical Analysis

Statistical analyses were performed using GraphPad Prism software (version 9.3.1). To check the normality of the distributions, all experimental data without primary analysis of the mRNA-seq data were evaluated with the Shapiro–Wilk normality test. Subsequently, nonparametric tests were used for all data. Comparisons of three groups were performed using Kruskal–Wallis H tests (one-way ANOVA of ranks) followed by a two-stage Benjamini, Krieger, and Yekutieli FDR procedure as a post hoc test. A *p*-value less than 0.05 was considered to indicate statistical significance.

## 3. Results

### 3.1. Overview of Gene Expression Profiling by Fasting-Refeeding

**Figure 2:**
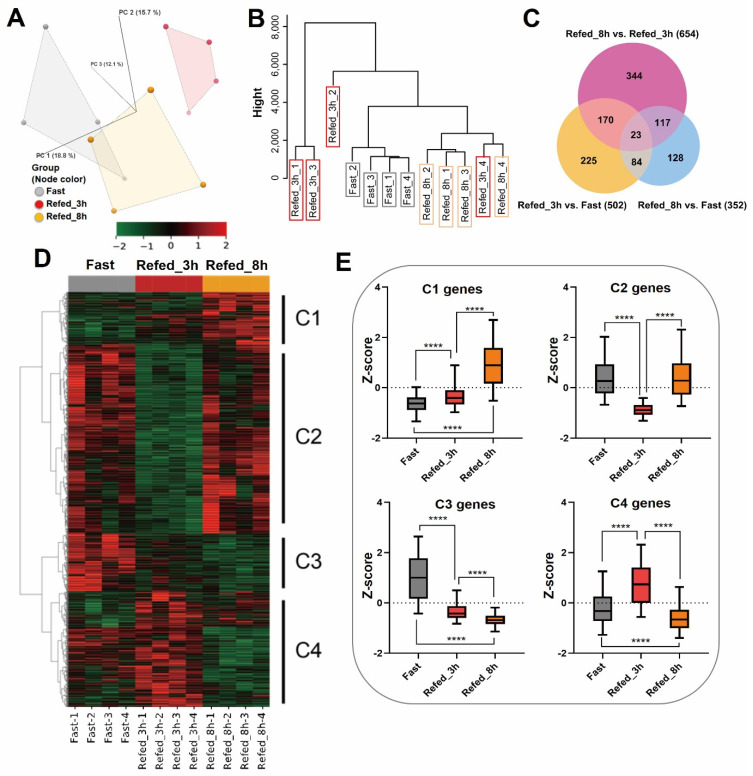
Gene expression profile of muscle tissues during fasting-refeeding conditions in zebrafish. (**A**) PCA plot and (**B**) cluster dendrogram depict similarities between samples. (**C**) Venn diagram shows the number of DEGs between three groups (Refed_8h vs. Refed_3h, Refed_3h vs. Fast, and Refed_8h vs. Fast). (**D**) Heat map represents expression of the 1091 DEGs. Values in the rows are z-scores. (**E**) Box plot shows expression patterns in each cluster on the heat map. The box indicates the first to the third quartile and the line indicates the median. The whiskers show the box with 5–95 percentile. **** *p* < 0.0001.

The mRNA-sequencing analysis was performed in three groups with different feeding conditions: 24 h fasting (Fast), refeeding after 3 h (Refed_3h), and refeeding after 8 h (Refed_8h). The PCA and cluster dendrogram of total gene expressions separated the Refed_3h from the other time points, and the Refed_8h had a similar expression pattern to the Fast condition (Figure 2A,B). The analysis of differentially expressed genes (DEGs) identified a total of 1091 expressed genes in three conditions. The Venn diagram (Figure 2C) exhibited 23 commonly shared DEGs between the three comparison groups. In addition, heat map and clustering analyses (Figure 2D) distinguished four gene-expression clusters that showed the following profiles. Clusters 1 (C1) and 3 (C3) were composed of genes with mutually inverse expression patterns between the fasting and the refeeding groups. Similarly, clusters 2 (C2) and 4 (C4) were composed of genes with opposite expression patterns. C2 genes, affected by the Refed_3h condition, were downregulated relatively to the other two conditions, while C4 genes, affected by Refed_3h, exhibited an inverse expression pattern (Figure 2E). These results indicate that the three groups show different gene expression patterns, and each group drives different biological processes. Furthermore, the correlation of gene expressions within C1 vs. C3 and C2 vs. C4 was mutually inverse.

### 3.2. Enrichment Analysis of DEG Profiles Affected by the Fasting-Refeeding

**Figure 3:**
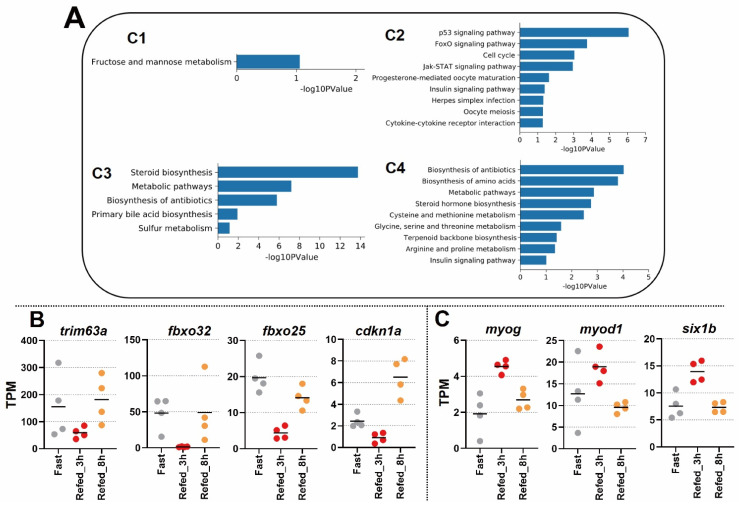
Enrichment analysis in KEGG pathway for gene clusters with confirmed expression values of individual samples. (**A**) Bar plots represent the analysis of significantly enriched pathways in each cluster. (**B**) Individual samples with TPM values of genes: *trim63a*, *fbxo32*, *fbxo25*, and *cdkn1a*. The bar indicates average mean. (**C**) Individual samples with TPM values of genes: *myog*, *myod1*, and *slx1b.* The bar indicates average mean.

To assess gene sets that may be associated with the biological pathways in each cluster (C1–C4) from Figure 2C, we performed a pathway analysis using the Kyoto Encyclopedia of Genes and Genomes (KEGG) together with the DAVID web tool. The results showed that the gene set of C1 was enriched in a pathway of the fructose and mannose metabolism (Figure 3A). The gene set of C2 was enriched in the p53 signaling pathway, FoxO signaling pathway, cell cycle, Jak-STAT signaling pathway, progesterone-mediated oocyte maturation, insulin signaling pathway, herpes simplex infection, oocyte meiosis, and cytokine-cytokine receptor interaction. The gene set of C3 was enriched in groups of genes related to steroid biosynthesis, metabolic pathways, biosynthesis of antibiotics, primary bile acid biosynthesis, and sulfur metabolism. Finally, the gene set of C4 was enriched in the following pathways, such as the biosynthesis of antibiotics; biosynthesis of amino acids; metabolic pathways; steroid hormone biosynthesis; cysteine and methionine metabolism; glycine, serine, and threonine metabolism; terpenoid backbone biosynthesis; arginine and proline metabolism; and the insulin signaling pathway. In particular, the fasting condition of the C2 showed a high gene-expression value of the fasting proteolytic system, such as markers of muscle atrophy *trim63a*, *fbxo32*, and *fbxo25* (Figure 3B). In addition, p21 (known as *cdkn1a*), being involved in p53 signaling, was upregulated at Refed_8h but was downregulated at Refed_3h (Figure 3A,B). We focused on a group of genes whose expression had rapidly increased after 3 h of the refeeding (Refed_3h); some genes associated with muscle development were enriched, e.g., *myog*, *myod1*, and *six1b* (Figure 3C). These results suggest that the opposite biological pathways of catabolic and anabolic metabolism were activated in fasting and refeeding conditions.

### 3.3. Identification of Differentially Expressed Genes in Zebrafish Skeletal Muscle Affected by Fasting-Refeeding

**Figure 4:**
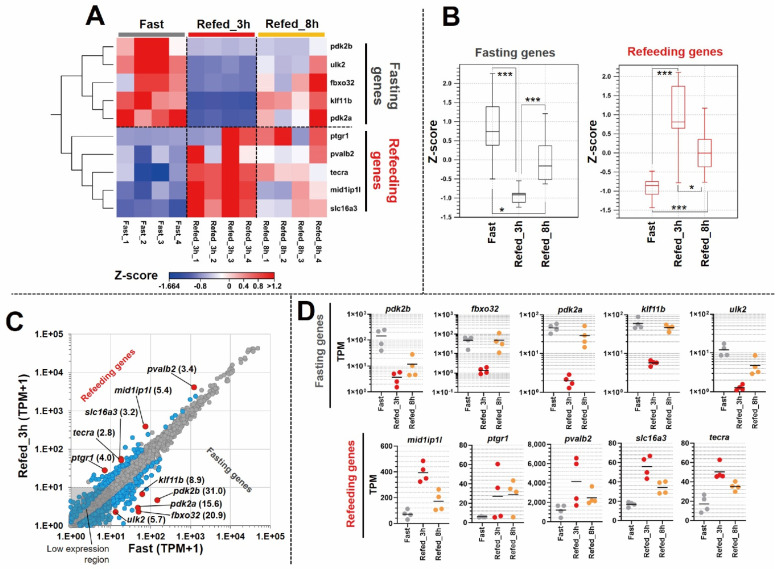
Identification of most affected genes in muscle tissues by fasting-refeeding. (**A**) Heat map reflects expression values as z-score for 10 genes (5 genes of each condition) identified as most responsive genes to fasting-refeeding conditions. Each group (Fast, Refed_3h, Refed_8h) is represented by 4 individual samples. (**B**) Box plot depicts a z-score of 10 genes in each group. The box indicates the first to the third quartile, and the line indicates the median. The whiskers show the box with 5–95 percentile. (**C**) Scatter plot reflects averages of TPM + 1 values of each gene, shown as red dots. Numbers in parentheses indicate the fold change between the Fast and Refed_3h groups. Gray dots indicate non-DEGs; light blue dots indicate DEGs as FDR *p*-value < 0.05. (**D**) Plot graph represents TPM values of individual samples with “fasting genes” and “refeeding genes”. The TPM values of fasting genes are represented by Log10 notation. The bar indicates average mean. * *p* < 0.05; *** *p* < 0.001.

Following the mRNA-sequencing, we performed a bioinformatics analysis to identify some genes that were affected by fasting-refeeding. Notably, gene clustering revealed that C2 and C4 had an immediate response to both Fast and Refed_3h conditions. Therefore, the normalized TPM values of the DEGs within C2 and C4, from the Fast vs. Refed_3h comparison, were processed by additional algorithmic analyses. The five most expressed genes from each group (Fast and Refed_3h) were visualized by heat map, box plot and scatter plot (Figure 4A–C). Interestingly, those five genes, which were most upregulated in the Fast group (*pdk2b*, *fbxo32*, *pdk2a*, *klf11b*, and *ulk2*), were downregulated in the Refed_3h group; therefore we named them as “fasting genes”. Furthermore, those five genes with dramatic upregulation in the Refed_3h group (*mid1ip1l*, *ptgr1*, *pvalb2*, *slc16a3*, and *tecra*) were downregulated in the Fast group; therefore we named them as “refeeding genes”. Particularly, *pdk2b* was upregulated 31 fold in the Fast group compared to the Refed_3h group. Similar to these results, consistent data were obtained from the plot graph (Figure 4D) evaluating the TPM values of an individual sample and gene. Following the expression pattern assessment, we analyzed the functions of those genes using publicly available databases (UniProtKB and ZFIN) (Table S2). The following predicted functions, such as glucose homeostasis, autophagy, and ubiquitination were related to the genes affected by the fasting condition. On the other hand, predicted functions, such as lipid and lactate metabolism were related to the genes affected by the refeeding condition. These results suggest the dramatic expression changes of genes related to various metabolic pathways by fasting-refeeding.

### 3.4. Novel Findings of Gene Length Associated with Refeeding

**Figure 5:**
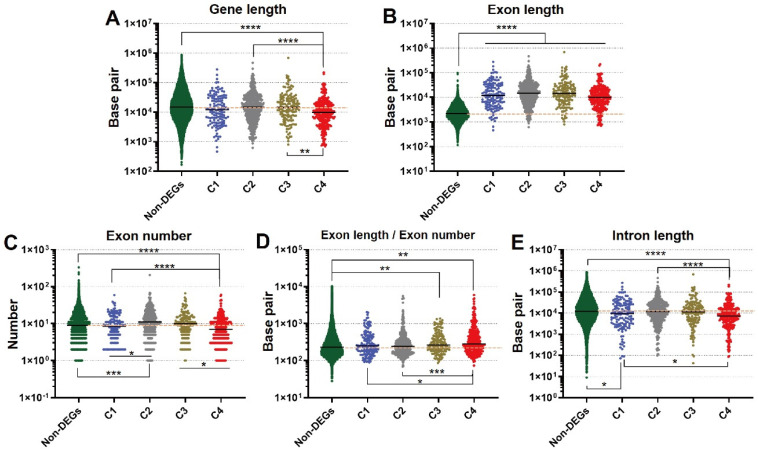
Analysis of gene length, exon length, and exon number in response to refeeding. The graphs show gene length (**A**), exon length (**B**), exon number (**C**), exon length/exon number (**D**), and intron length (**E**). The bar indicates the average mean and the orange dashed line shows the average mean of the non-DEGs. * *p* < 0.05, ** *p* < 0.01, *** *p* < 0.001, **** *p* < 0.0001, respectively.

Previous studies have suggested that gene length is involved in life processes and diseases [24,25,26,27,28,29,30,31,32]. However, it is unclear how genes involved in fasting-refeeding conditions are associated with gene length. Therefore, we analyzed the length distribution of the gene group that corresponded to each cluster and observed that genes of C4 were significantly shorter than the non-DEGs, genes of C2, and genes of C3 (Figure 5A). In particular, the C4 genes had a significantly lower number of exons than non-DEGs, genes of C1, C2, and C3 (Figure 5C). Additionally, the average intron length of C4 genes was significantly lower than that of non-DEGs, genes of C1, and genes of C2 (Figure 5E). On the other hand, exon length and length per exon of C4 genes were significantly longer than those in other clusters (Figure 5B,E). These results indicate that the C4 genes have a long exon length, however, they have a shorter intron and a smaller exon number, resulting in a shorter length of genes affected by refeeding.

## 4. Discussion

Our results of the enrichment analysis showed that gene expressions associated with the FoxO signaling pathway were increased under the fasting condition. In particular, the expressions of *fbxo32* and *trim63a* in cluster 2 were increased as a response to Fast and Refed_8h, and were decreased as a response to Refed_3h (Figure 3A,B). These genes are considered as “atrogenes” that relate to E3 ubiquitin ligase. *TRIM63* and *FBXO32* especially, which encode MuRF1 and MAFbx, respectively, are widely known as master regulators of skeletal muscle atrophy in rodents and humans [33,34,35]. Those genes have reportedly been upregulated by fasting for 10 days in teleosts [36] or for 24 h in rodents [37]. Moreover, the present study obtained similar results to previous studies; thus, these findings may provide evidence that fasting induces skeletal muscle atrophy. This suggests that zebrafish skeletal muscle is affected by fasting under a similar molecular mechanism as in mammals. Additionally, in the conditions of Fast and Refed_8h, both *trim63a* and *fbxo32* exhibited similar expression, respectively (Figure 3B). Our data indicates that the molecular signal of proteolysis via the FoxO signaling pathway in the skeletal muscle might have reached the upper limit of saturation within the short-term period of the 8 h. On the other side, muscle protein synthesis might have increased within 3 h after the refeeding, which is supported by data from the enriched pathway analysis (amino acid metabolism) (C4 in Figure 3A). Interestingly, muscle degradation and protein recycling by the ubiquitin–proteasome system seemed to be recovered after 8 h since the refeeding, as a consequential biological process in the protein turnover. It has been reported that abnormalities of the ubiquitin–proteasome system together with increased expressions of MuRF-1 and MAFbx are associated with muscle atrophy in diseases such as cancer cachexia, sarcopenia of aging, chronic kidney disease, diabetes, and chronic obstructive pulmonary disease [38]. Moreover, the intake of fatty acids or proteins also alters the activity of the ubiquitin–proteasome system [38]. In experiments using a rat model of hindlimb immobilization, it was reported that supplementation of leucine and branched-chain amino acids had prevented the accumulation of ubiquitinated proteins; in contrast, upregulations of MuRF-1 and MAFbx in the soleus muscle had minimized the loss of muscle mass caused by the immobilization [39]. Thus, the responses of the ubiquitin–proteasome system to disease or nutrient metabolisms are closely related in mammals, whereas zebrafish may represent a similar system. However, our zebrafish model shows that gene expressions related to the ubiquitin–proteasome system exhibited large fluctuations in the short-term periods after feeding. It is expected that further subdivision of the time-period after the refeeding will help to elucidate the molecular mechanisms more precisely. Taken together, this study reveals the capability of a zebrafish model to explore the molecular mechanisms of the ubiquitin–proteasome system in disease or nutrient metabolisms, while being more pragmatic than a mammalian model.

Next, the expressions of C2 genes with enrichment in p53 and p21 signaling pathways were upregulated in the fasting condition (Figure 3A). A previous study in mice showed that p21, being involved in p53 signaling, is upregulated after a 48 h fast, suggesting that p21 is associated to elevated energy expenditure, depletion of fat stores, and premature activation of protein catabolism in mice muscle [40]. Taken together, zebrafish muscle tissues seem to have p21-related mechanisms similar to those within murine muscles during the fasting condition.

The gene expressions of cluster 4 were inversely correlated with the expressions of the C2 genes. Genes involved in myogenesis were enriched in C4. Our results showed that after the 24 h fasting, *myog* and *myod1* expressions were upregulated in the Refed_3h group (Figure 3C). Consistent with our results, the gene *myog* was reported to have an increased expression in zebrafish skeletal muscle 6 h after refeeding, though there were differences in the fasting-refeeding experimental setup (e.g., time point and food type) [23]. However, in the previous study, *myod1* was not noted to be upregulated [23]. This discrepancy in results may be caused by differences in experimental conditions. For instance, Seiliez et al. reported on zebrafish that underwent fasting for 72 hours with a following refeeding *ad libitum* and muscle tissues were harvested at 0.5, 2, 6, and 24 h after the refeeding [23]. Our results may have captured a more immediate response because we observed gene expression 3 h after refeeding. In addition, differences in sampling sites may have affected the results. In order to resolve these differences in the future, it might be necessary to establish detailed and consistent experimental methods.

Our data also showed that *six1b* was upregulated only 3 h after refeeding (Figure 3C). There are two orthologs in zebrafish, *six1a* and *six1b*, which have been reported to have important functions in normal muscle formation during embryonic development [41]. The results in this study showed that *six1b* changed immediately during the response to the refeeding. Therefore, an increased expression of *six1b* caused by refeeding may be involved in the normal muscle formation of adult zebrafish.

Next, we identified several genes that were dramatically affected by a fasting-refeeding protocol. In total, 10 genes (from C2 and C4) exhibited high expression values and high fold changes within Fast or Refed_3h conditions (Figure 4A–D). To date, there have been no reports of those genes being affected by fasting and refeeding in zebrafish skeletal muscle. Therefore, these findings provide new insights for researchers in nutrition, internal medicine, etc., to use zebrafish as a model. For instance, these 10 genes could be utilized in studies as responding markers to new nutrients or compounds for muscle treatment. On the other hand, new cases of people suffering obesity have recently increased, as the present day may be considered as an era of satiety. Additionally, sarcopenic obesity among adults is rapidly increasing worldwide [42]. Sarcopenia is a condition characterized by a loss of muscle mass, strength, or physical function, while bearing a potential to induce a frequency of metabolic disorders, cardiovascular disease, and mortality [42]. Although the present study used a zebrafish model, we identified an overall mRNA-expression profile and the 10 most affected genes. Therefore, our findings could be useful in treatment development and preventive strategies for sarcopenic obesity.

Gene length has been reported to be associated with biological regulatory processes. Previous studies have suggested that carcinogenesis and other multigenic diseases, embryonic development and neuronal processes are associated with the presence of long genes [24,25,26,27,28,29,30,31,32]. In addition, bioinformatics analysis indicated that short genes are mainly involved in the immune system and skin formation [43]. These studies lead to the hypothesis that genes with long transcripts are primarily associated with early developmental function, and genes with small transcripts play an important role in routine functions [43]. Although the relationship between gene length and biological process is yet to be fully understood, we hypothesize that this mechanism is associated with genes that respond to fasting-refeeding. According to the results, the analysis of gene length in the DEGs cluster showed that C4 contained significantly shorter genes (Figure 5A), and the genes in C4 had fewer exons (Figure 5C). The representative genes from C4 may have been efficiently designed during evolution to provide a rapid biological response to refeeding in daily life, which is supported by the hypothesis of the previous study [43]. In addition, chromosomal regions radiate within the nucleus including those chromosomes with a predominant peripheral location (heterochromatin), and chromosomes with active genes located inside the nucleus (euchromatin) [44]. We propose that some of the nutritionally regulated short length genes may be located in euchromatin regions with an efficient transcription under nutritional response. Taken together, these results provide new insights into the role gene length has on gene expression.

The effect of dietary restrictions on murine lifespan depends on species, strain, and age at the start of an experiment, with both negative and positive effects reported [45,46]. Therefore, it is necessary to consider whether these differences also occur in zebrafish. Our results were obtained from the single fasting-refeeding response, and it is unclear whether these results will correspond to those after a multiple fasting-refeeding approach. Apart from that, the data on gene length were obtained by bioinformatics analysis using a small number of samples, leaving the task of clarifying the actual biological mechanism and significance. Therefore, further in vitro and in vivo investigation is needed to confirm the molecular findings predicted by this bioinformatics analysis.

## 5. Conclusions

This study focused on the transcriptome response of fasted zebrafish skeletal muscle tissue. The novel findings in this study, acquired by reliable methods of bioinformatics analysis, provide the use of new gene sets in a zebrafish model with dynamic responses to the fasting condition—“fasting genes”, and the refeeding condition—“refeeding genes”. Furthermore, this bioinformatics analysis discovered new views on gene length in the regulation of gene expression under fasting-refeeding conditions. Based on these results, it is expected that zebrafish will provide a new experimental model for fasting-refeeding response and, hence, further significance as an animal model. Future studies may need to continuously assess short- and long-term molecular changes due to fasting or refeeding and link them to positive and negative effects on diseases and other conditions, as well as being metabolic markers.

## Data Availability

The mRNA-seq data as FASTQ files and expression browser as table data have been deposited in the “Gene Expression Omnibus (https://www.ncbi.nlm.nih.gov/geo/; accessed on 22 April 2022)” under accession number: GSE201273.

## Supplementary Materials

The following supporting information can be downloaded at: https://www.mdpi.com/article/10.3390/nu14112239/s1, Table S1: Expression values of all genes; Table S2: Predicted function and fold-change of the top five genes in each condition.
